# Hints for Nurses

**Published:** 1874-02

**Authors:** 


Hints for Nurses.—The following sensible suggestions are from the pen of Florence Nightengale:

Conciseness and decision are, above all things, necessary with the sick. Let your thoughts expressed to them be concisely and decidedly expressed. What doubt and hesitation there may be in your own mind must never be communicated to theirs, not even (I would rather say especially) in little things. Let your doubt be to yourself, your decision to them. People who think outside their heads, the whole process of whose thought appears, like Homer's, in the act of secretion, who tell everything that led them towards this conclusion and away from that, ought never to be with the sick.

Irresolution is what all patients most dread. Rather than meet this in others, they will collect all their data, and make up their minds for themselves. A change of mind in others, whether it is regarding an operation or rewriting a letter, always injures the patient more than the being called upon to make up his mind to the most dreaded or difficult decision. Further than this, in very many cases the imagination in disease is far more active and vivid than it is in health. If you propose to the patient change of air to one place one hour, and to another the next, he has, in each case, immediately constituted himself, in imagination, the tenant of the place, gone over the whole premises in idea, and you have tired him as much by displacing his imagination as if you had actually carried him over both places.

